# Childhood violence and adult chronic pain among indigenous Sami and non-Sami populations in Norway: a SAMINOR 2 questionnaire study

**DOI:** 10.3402/ijch.v75.32798

**Published:** 2016-10-31

**Authors:** Astrid M. A. Eriksen, Berit Schei, Ketil Lenert Hansen, Tore Sørlie, Nils Fleten, Cecilie Javo

**Affiliations:** 1 Sami National Centre for Mental Health and Substance Use (SANKS) Finnmarkssykehuset HF, Karasjok, Norway; 2 Faculty of Health Science, Oslo and Akershus University College of Applied Science, Oslo, Norway; 3 Department of Community Medicine, Institute of Health and Society, University of Oslo, Oslo, Norway; 4 Department of Public Health, NTNU, Trondheim, Norway; 5 Department of Obstetrics and Gynecology, St. Olav's Hospital, Trondheim University Hospital, Trondheim, Norway; 6 Regional Centre for Child and Youth Mental Health and Child Welfare, UiT – The Arctic University of Norway, Tromsø, Norway; 7 Department of Clinical Medicine, UiT – The Arctic University of Norway, Tromsø, Norway; 8 Department of Mental Health and Substance Abuse, University Hospital of North Norway, Tromsø, Norway; 9 Department of Community Medicine, UiT – The Arctic University of Norway, Tromsø, Norway

**Keywords:** chronic pain, childhood emotional, physical, sexual violence, child abuse, ethnicity, Sami, indigenous, SAMINOR 2 study, Norway

## Abstract

**Background:**

Internationally, studies have shown that childhood violence is associated with chronic pain in adulthood. However, to date, this relationship has not been examined in any indigenous population.

**Objective:**

The main objectives of this study were to investigate the association between childhood violence and reported chronic pain, number of pain sites and the intensity of pain in adulthood in indigenous Sami and non-Sami adults, and to explore ethnic differences.

**Design:**

The study is based on the SAMINOR 2 questionnaire study, a larger population-based, cross-sectional survey on health and living conditions in multiethnic areas with both Sami and non-Sami populations in Mid- and Northern Norway. Our study includes a total of 11,130 adult participants: 2,167 Sami respondents (19.5%) and 8,963 non-Sami respondents (80.5%). Chronic pain was estimated by reported pain located in various parts of the body. Childhood violence was measured by reported exposure of emotional, physical and/or sexual violence.

**Results:**

Childhood violence was associated with adult chronic pain in several pain sites of the body regardless of ethnicity and gender. Childhood violence was also associated with increased number of chronic pain sites and higher pain intensity compared to those not exposed to childhood violence. However, among Sami men, this association was only significant for pain located in chest, hips/legs and back, and non-significant for increased number of chronic pain sites (adjusted model), and higher pain intensity.

**Conclusion:**

Respondents exposed to childhood violence reported more chronic pain in several parts of the body, increased number of chronic pain sites and more intense pain in adulthood than respondents reporting no childhood violence. However, among Sami men, this association was weaker and also not significant for increased number of chronic pain sites and higher pain intensity.

Interpersonal violence against children is acknowledged as a major global health problem by the World Health Organization. The concept includes psychological, sexual and physical forms of violence (1). International studies have indicated a higher prevalence of interpersonal violence in indigenous populations compared to the dominant group in their country (2–4). Therefore, interpersonal violence is considered a major public health problem also among indigenous peoples. As the association between childhood adversities and health status has been sparsely focused on in medical and psychological research among Arctic indigenous groups, a greater focus on research that may increase our knowledge for these populations is warranted. This study aims to fill the knowledge gap of the relationship between childhood violence and adult health among the indigenous Sami and non-Sami in Mid- and Northern Norway.

In a previous article, we have shown that childhood violence (emotional, physical and sexual) is more commonly reported by the Sami than by the non-Sami (2). Health effects of violence may be devastating: childhood violence is associated with poorer mental health (5–10) and several studies have documented that children exposed to interpersonal violence have increased risk of chronic pain conditions in adulthood, such as headache, pelvic pain, muscular and abdominal pain (11–15).

Pain is a global and complex phenomenon with biological, psychological and sociocultural aspects intertwined. Chronic pain can be tied to both well-known somatic disorders and conditions, but also to mental and psychological conditions. It has been found that a history of childhood abuse or violence, being female, older age and lower socio-economic status (SES) are generally associated with chronic pain (16). Psychosocial factors such as depression sleep problems and lifestyle factors like unemployment are also found to associate with chronic pain (17–20). Moreover, studies have found that an increased number of pain sites seem to predict a future disability pension (15,21).

Ethnic differences in reported chronic pain have been reported: studies from the UK reported chronic pain to be more prevalent among ethnic minority groups (22). In studies from the USA, self-reported chronic pain was higher in Hispanic and Black ethnic groups than among non-Hispanic Whites. Moreover, indigenous populations like American Indians and Alaska Natives were more prone to chronic pain conditions such as rheumatic diseases, headache, low back pain and other conditions associated with chronic pain (23–25). Most studies have used data from public statistics with no association to other conditions, such as childhood abuse, violence and harassment. However, one study among American Indian tribes reported a strong association between post-traumatic stress disorder (PTSD) and bodily pain (26). Studies on chronic pain among the aboriginals in Canada are sparse (25). However, figures from Statistics Canada show that Canadian aboriginals reported a higher prevalence of chronic pain compared to the majority population (27). A considerable body of literature suggests that diverse biological, psychological and sociocultural mechanisms contribute to disparities by race and ethnicity. A higher prevalence of risk factors for illness, such as lower SES, poor health habits and inadequate access to health care services, predisposes minority groups to suffer a higher burden of pain (25). However, more detailed studies exploring the association between childhood violence and chronic pain in these groups are warranted.

The indigenous people of Norway are the Sami. The traditional living area of the Sami people is the Arctic regions of Norway, Sweden and Finland, and the Russian Kola Peninsula. Their traditional way of living has been semi nomadic (reindeer herding). Today, less than 10% of the Sami are reindeer herders. Due to historical colonization and oppression, many Sami abandoned their Sami identity with a resulting loss of Sami language and culture (28). Like other indigenous populations, the Sami people have a traumatic history of various types of social injustice and oppression (29,30).

Literature on reported chronic pain among the Sami is sparse. A recent population-based study among Sami and Norwegian adolescents, which estimated musculoskeletal pain (headache, neck/shoulder, back and arm/knee/leg) during the last 12 months and number of pain sites, found no major ethnic differences (31). Anxiety/depression symptoms were the dominant factor associated with musculoskeletal pain followed by negative life events and school-related stress. The Norwegian Institute of Public Health found that Sami reported less chronic pain than Norwegians (data from the SAMINOR study, 2003/2004). A master thesis investigating chronic pain among Sami, Kven and Norwegians (36–65 years old) found that Sami who lived in the highland had lower odds for chronic pain compared to Norwegians in coastal areas (32). Contrary to the findings mentioned above, Sami reported general abdominal pain more often than the majority Norwegian population. More stomach symptoms among the Sami were found after consuming milk, suggesting a higher prevalence of milk intolerance among the Sami than the majority population (33). Other studies among Sami in Finland and Russia confirm the higher prevalence of lactose intolerance compared to the majority populations (34,35). Moreover, a study based on SAMINOR data reported a higher prevalence of angina pectoris (heart cramp) among the Sami compared to the non-Sami (36).

Culture may affect pain perception (37). Results from a recent qualitative study in northern Norway indicate that patterns of how pain is interpreted and expressed in adult life are established in childhood, influenced by parenting styles and values (38). While Sami communities traditionally have emphasized the value of enduring discomfort and pain without complaining, many Western, including Norwegian, cultures encourage children to immediately seek comfort and tell an adult what hurts (38). In a study by Javo et al., it was found that Sami and Norwegian parents differed in their parenting and that Sami and Norwegian children reacted differently to a tougher disciplinary parenting style (39,40). In their child rearing, Sami parents placed a strong value on inner strength, hardiness and the child's ability to withstand hardships (41).

To our knowledge, the association between reported childhood violence and adult chronic pain has not been previously investigated, neither in a Sami population nor in a Sami versus non-Sami population (search in data bases PubMed, Medline). Thus, the aims of this study were to investigate the association between interpersonal violence experienced in childhood and adult chronic pain within a Sami and non-Sami population in Norway, and to explore possible ethnic differences in the association between childhood violence and chronic pain in adulthood. In both ethnic groups, we expected a positive association between childhood violence and adult chronic pain, and that those respondents who reported exposure to childhood violence would have a higher number of chronic pain sites compared to respondents who were not exposed to childhood violence. However, we expected this association to be weaker among the Sami respondents compared to the non-Sami respondents. We assumed that due to cultural differences in child-rearing, where Sami parents appear to place a stronger value on the child's inner strength and tolerance for unpleasant experiences, the association between reported childhood violence and adult pain would be less prominent in the Sami respondents, especially in men.

## Materials and methods

This study is an integral part of a larger questionnaire-based population study on health and living conditions in areas with both Sami and Norwegian populations, the SAMINOR 2 questionnaire study. The previous population-based study, the SAMINOR 1 study, was conducted in 2003/2004 and has been described in detail in a previous paper (42). The SAMINOR 2 questionnaire study was both designed as a follow up study of issues addressed in this first SAMINOR study and was expanded to include additional health issues, such as mental health and interpersonal violence. The SAMINOR 2 questionnaire study is described more fully in a recent paper (43).

### Sample

**Fig. 1:**
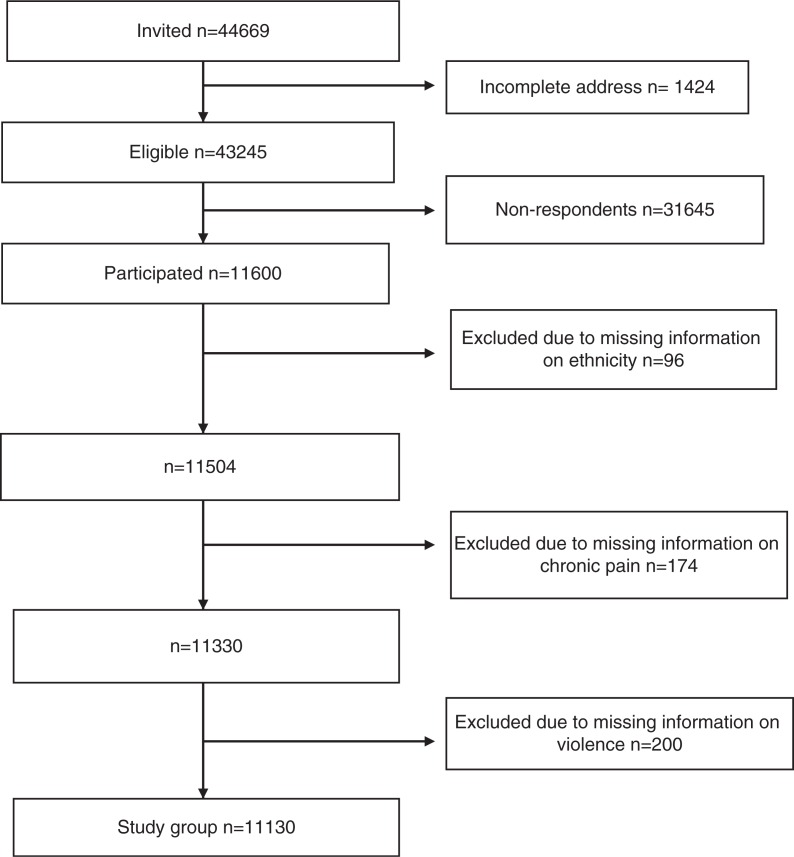
Flow chart of inclusion in the study population.

All inhabitants aged 18–69 years registered in the Central Population Registry in selected municipalities with Sami and non-Sami populations received an invitation letter to participate in the investigation. A questionnaire was sent by post to 44,669 persons; 1,424 questionnaires were returned unopened and hence classified as technically missing, leaving 43,245 persons eligible for the study. Among these, 11,600 returned a filled-out questionnaire, yielding a participation rate of 27%. Further methodological details are described elsewhere (43). In the present study, 96 respondents were excluded due to missing information on ethnicity, 174 respondents were excluded due to missing information on chronic pain and 200 respondents were excluded due to missing information on violence, leaving 11,130 as the study sample (Figure 1): 2,167 Sami (19.5%) and 8,963 non-Sami (80.5%).

### Chronic pain

Chronic pain was measured by the question “Have you during the last year been affected with pain and/or stiffness in muscles and/or skeleton which have lasted for at least 3 months?” The response options were “Yes” and “No.” Further, the respondents were asked to indicate which part(s) of the body were affected with the following response options: “Neck, shoulders,” “Arm, hands,” “Upper part of the back,” “Lumbar/Lower part of the back,” “Hips, legs,” “Head,” “Chest,” “Stomach,” “Pelvic” and “Other places.” Positive answer to one or more of the body sites were merged into one category: “Any pain.” For each response option, the respondents were asked to indicate the intensity of the pain with the following response options: “Not affected,” “Somewhat affected” and “Strongly affected.” Those answering “Somewhat affected” and “Strongly affected” were merged into the category “Yes, affected,” and defined as the chronic pain group. The remaining study group was defined as the no-chronic pain group. Further, in the logistic regression analysis (Tables IV and V), pains located in the upper and lower parts of the back were merged into one category: “Back pain.” Correspondingly, pains located in the stomach and pelvic were merged into one category: “Stomach/pelvic pain.”

### Classifying ethnicity

Ethnicity was assessed using responses to the following questions: “What language does/did you use at home?,” “What language did your parents use at home?,” “What language did your grandparents use at home?” and “What do you consider yourself to be?” Response options were “Norwegian,” “Sami,” “Kven” and “Other.” Participants who chose “Sami” in response to any of the three first questions, in addition to self-identification as a Sami person, were classified as belonging to the Sami ethnic group. Norwegians, Kvens (descendants of Finnish immigrants) and Others were defined as non-Sami. The vast majority of this group was ethnic Norwegians.

### Questions on violence

Three sections of questions in the questionnaire addressed experience of emotional, physical and/or sexual violence. Participants who responded positively to the question “Have you experienced that someone systematically and over time has tried to repress or humiliate you?” were classified as “having experienced emotional violence.” Participants who responded positively to the question *“*Have you been exposed to physical assault/abuse?” were classified as “having experienced physical violence.” Participants who responded positively to the question “Have you been exposed to sexual assault?” were classified as “having experienced sexual violence.” These three categories of violence were then merged into one category: “Any violence.” Participants who responded positively to having experienced any type of violence (sexual, physical and/or emotional) were defined as “having experienced any violence.” Moreover, respondents were asked to indicate whether the violence had occurred in childhood and/or in adulthood with the following alternatives: “Yes, as a child,” “Yes, as an adult” and “Yes, in past 12 months.” Participants who reported having experienced emotional, physical and/or sexual violence as a child were defined as having been exposed to the different types of violence in childhood.

### Other variables

Background information such as the level of education and family/household income was collected from the questionnaire. Age and gender were imputed from the Statistics Norway (SSB). Age was classified by year of birth and further categorized into three age groups: 18–34, 35–49 and 50–69 years. Education was classified as number of years at school and was categorized into four groups: primary school (≤9 years), high school (10–12 years), higher university or college education (13–15 years) and university educations (≥16 years). Family/household income per year was categorized into three groups: low (<150,000–300,000 NOK (NOK=Norwegian krone), medium (301,000–600,000 NOK) and high (>600,000 NOK). Both education level and family income level may be used as a proxy for SES. We decided to use the former in our study. Family income is presented in Table I for the sake of information only. “Any other specific symptom” was created based on a positive response to the following question: “Do you have, or have you had, diabetes, high blood pressure, angina pectoris (heart cramp), heart attack, psychological problems, chronic bronchitis, asthma, eczema, psoriasis, multiple sclerosis and/or Bechterew's disease?”

**Table I:** Background characteristics, reported childhood violence and adult chronic pain in the Sami and non-Sami population by gender

	Women (n=6,210)					Men (n=4,920)				
				
	Sami (n=1,226)	%	non-Sami (n=4,984)	%	p^a^	Sami (n=941)	%	non-Sami (n=3,979)	%	p^a^
Age					<0.001					0.987
18–34	307	25.0	1,000	20.1		150	15.9	628	15.8	
35–49	368	30.0	1,707	34.2		272	28.9	1,159	29.1	
50–69	551	44.9	2,277	45.7		519	55.2	2,192	55.1	
Education (years)					<0.001					0.172
Primary school (≤9)	136	11.2	629	12.7		186	20.0	703	17.8	
High school (10–12)	223	18.4	1,249	25.3		290	31.1	1,225	31.1	
College/university (13–15)	310	25.6	1,329	26.9		215	23.1	1,032	26.2	
University (≥16)	540	44.7	1,731	35.1		241	25.9	981	24.9	
Income					<0.001					<0.001
Low	197	16.1	681	13.7		166	17.6	522	13.1	
Medium	512	41.8	1,833	36.8		352	37.4	1,416	35.6	
High	468	38.2	2,318	46.5		402	42.7	1,957	49.2	
Childhood violence					<0.001					<0.001
Emotional	252	20.6	630	12.6	<0.001	196	20.8	480	12.1	<0.001
Physical	146	11.9	469	9.4	0.009	129	13.7	285	7.2	<0.001
Sexual	207	16.9	578	11.6	<0.001	46	4.9	145	3.6	0.076
Any violence	382	31.2	1,072	21.5	<0.001	264	28.1	639	16.1	<0.001
Any chronic pain	647	52.8	2,747	55.1	0.140	456	48.5	1,910	48.0	0.801

a Comparing Sami and non-Sami by Pearson chi-squared test.

### Ethics

The data collection and storage of data were approved by the Norwegian Data Protection Authority (Datatilsynet). Written informed consent was obtained from all the participants. The study was approved by the Regional Committee for Medical and Health Research Ethics of Northern Norway (REK -Nord) and SSB.

### Statistical analysis

IBM SPSS Statistics Version 22.0 for Windows was used to conduct statistical analyses. Frequencies and cross tabulations gave the distribution of socio-demographic variables, childhood violence and chronic pain. Chi-square test was used for the comparison between the group who reported childhood violence and the group reporting no childhood violence, by ethnicity and gender. Level of significance was set to 5%. Stratified logistic regression analyses by gender were performed to assess the association between any childhood violence and any chronic pain. These analyses were separate and independent, using enter modelling. In addition, we stratified the analysis on the different pain sites. In model 1, ethnicity was included. In model 2, ethnicity, age and education were included. In model 3, we also included any other specific symptoms. We investigated any interaction between childhood violence and ethnicity on the different dependent variables of chronic pain. The strength of the associations in the logistic models was presented with a 95% confidence interval (95% CI). We also conducted stratified analysis by the different types of violence on the association on any chronic pain. Bivariate analysis gave the mean number of chronic pain sites. Independent sample t-tests were conducted to explore any differences based on ethnicity and exposure to childhood violence. One-way analysis of variance (ANOVA) was conducted to explore differences between age groups and education groups. Furthermore, we assessed the association between any childhood violence and number of chronic pain sites. Interaction between any childhood violence and ethnicity was tested, as well as interaction between childhood violence and age groups on any chronic pain. Poisson regression analysis was performed to assess the association between any childhood violence and number of chronic pain sites, stratified by ethnicity and gender.

## Results

The proportion of young and long-educated women was higher for Sami than non-Sami, whereas there were no ethnic differences in age and educational level between Sami and non-Sami men (Table I). Sami respondents reported lower income than non-Sami respondents (Table I). Compared to non-Sami, stomach pain and pelvic pain were more frequently reported by Sami women, and chest pain and stomach pain were more frequently reported by Sami men (Table II). There were no ethnic differences in any chronic pain (Table I) or mean number of pain sites (Table VI). Among all, 6,167 (55.4%) reported any other specific symptom (somatic or psychological).

**Table II:** Respondents reporting chronic pain by childhood violence and total among Sami and non-Sami women

	Sami women (n=1,226) Any childhood violence			Non-Sami women (n=4,984) Any childhood violence			All Women (n=6,210)				
						
Chronic pain	Yes (n=382) n (%)	No (n=844) n (%)	p^a^	Yes (n=1,072) n (%)	No (n=3,912) n (%)	p^a^	Sami (n=1,226)	%	Non-Sami (n=4,984)	%	p^a^
Any pain	236 (61.8)	411 (48.7)	<0.001	666 (62.1)	2,081 (53.2)	<0.001	647	52.8	2,747	55.1	0.140
Neck, shoulders	196 (51.3)	308 (36.5)	<0.001	515 (48.0)	1,588 (40.6)	<0.001	504	41.1	2,103	42.2	0.490
Arms	138 (36.1)	228 (27.0)	0.001	384 (35.8)	1,111 (28.4)	<0.001	366	29.9	1,495	30.0	0.922
Back	117 (30.6)	166 (19.7)	<0.001	334 (31.2)	856 (21.9)	<0.001	283	23.1	1,190	23.9	0.559
Lumbar	152 (39.8)	218 (25.8)	<0.001	434 (40.5)	1,165 (29.8)	<0.001	370	30.2	1,599	32.1	0.200
Hips, leg	151 (39.5)	253 (30.0)	0.001	449 (41.9)	1,277 (32.6)	<0.001	404	33.0	1,726	34.6	0.267
Head	87 (22.8)	115 (13.6)	<0.001	249 (23.2)	573 (14.6)	<0.001	202	16.5	822	15.6	0.989
Chest	51 (13.4)	69 (8.2)	0.005	133 (12.4)	271 (6.9)	<0.001	120	9.8	404	8.1	0.058
Stomach	89 (23.3)	125 (14.8)	<0.001	192 (17.9)	407 (10.4)	<0.001	214	17.5	599	12.0	<0.001
Pelvic	52 (13.6)	56 (6.6)	<0.001	124 (11.6)	217 (5.5)	<0.001	108	8.8	341	6.8	0.017
Other	25 (6.5)	28 (3.3)	0.010	74 (6.9)	130 (3.3)	<0.001	53	4.3	204	4.1	0.717

a Comparing childhood violence history by Pearson chi-squared test.

Ethnic and gender differences in the reported prevalence of childhood violence are included in Table I.

### Childhood violence and chronic pain reports

Respondents reporting any childhood violence also reported more chronic pain in all body sites than respondents reporting no childhood violence regardless of gender and ethnicity (Tables II and III). However, among Sami men, the results did not reach a level of significance, although the pattern was similar to that of non-Sami men. Logistic regression analysis showed higher odds ratio (OR) for any chronic pain as adults when respondents reported childhood violence (Tables IV and V). Stratified analysis on the different types of violence showed a similar pattern with no major differences on the association between the different types of violence on any chronic pain (data not shown). The pattern of higher OR for any chronic pain as adults if the respondents reported childhood violence remained the same in the adjusted models. However, the results did not reach the level of significance among Sami men (Tables IV and V). In the stratified analysis on gender, interaction between any childhood violence and ethnicity on any chronic pain was not significant. However, in the adjusted models (models 2 and 3), the interaction among men was significant (p=0.023). In the stratified analysis on the different pain sites, interaction between any childhood violence and ethnicity on pain located in neck/shoulder was found among women (p=0.033), that is, the association between pain located in neck/shoulder and any childhood violence was stronger for Sami than non-Sami women (Table IV). However, in the adjusted models, the interaction was not significant. Among men, significant interaction between childhood violence and ethnicity was found on the association between pain located in arms/hands (p=0.017), neck/shoulder (p=0.006), head (p=0.034), stomach/pelvic (p=0.008) and other places (p=0.006). These associations were weaker and did not reach the level of significance for Sami men (Table V). However, among Sami men, a significant association between childhood violence was found on pain located in the back, hips/legs and chest.

**Table III:** Respondents reporting chronic pain by childhood violence and total among Sami and non-Sami men

	Sami men (n=941) Any childhood violence			Non-Sami men (n=3,979) Any childhood violence			All men (n=4,920)				
						
Chronic pain	Yes (n=264) n (%)	No (n=677) n (%)	p^a^	Yes (n=639) n (%)	No (n=3,340) n (%)	p^a^	Sami (n=941)	%	Non-Sami (n=3,979)	%	p^a^
Any pain	136 (51.5)	320 (47.3)	0.136	370 (57.9)	1,540 (46.1)	<0.001	456	48.5	1,910	48.0	0.801
Neck, shoulders	93 (35.2)	226 (33.4)	0.322	273 (42.7)	1,053 (31.5)	<0.001	319	33.9	1,326	33.3	0.737
Arms	72 (27.3)	166 (24.5)	0.214	198 (31.0)	715 (21.4)	<0.001	238	25.3	913	22.9	0.126
Back	54 (20.5)	103 (15.2)	0.053	124 (19.4)	443 (13.3)	<0.001	157	16.7	567	14.2	0.058
Lumbar	82 (31.1)	195 (28.8)	0.272	218 (34.1)	847 (25.4)	<0.001	277	29.4	1,065	26.8	0.098
Hips, leg	84 (31.8)	178 (26.3)	0.089	226 (35.4)	827 (24.8)	<0.001	262	27.8	1,053	26.5	0.390
Head	25 (9.5)	52 (7.7)	0.220	94 (14.7)	235 (7.0)	<0.001	77	8.2	329	8.3	0.932
Chest	31 (11.7)	58 (8.6)	0.087	67 (10.5)	195 (5.8)	<0.001	89	9.5	262	6.6	0.002
Stomach	30 (11.4)	70 (10.3)	0.362	82 (12.8)	243 (7.3)	<0.001	100	10.6	325	8.2	0.016
Pelvic	16 (6.1)	37 (5.5)	0.414	52 (8.1)	130 (3.9)	<0.001	53	5.6	182	4.6	0.171
Other	12 (4.5)	33 (4.9)	0.492	48 (7.5)	103 (3.1)	<0.001	45	4.8	151	3.8	0.164

a Comparing childhood violence history by Pearson chi-squared test.

**Table IV:** The association between any childhood violence and chronic pain in women

			Model 1^a^		Model 2^b^		Model 3^c^	
					
Women (n=6,210)	Crude OR	CI	OR	CI	OR	CI	OR	CI
Any chronic pain								
Any violence	1.5	1.3–1.7	1.5	1.3–1.7	1.7	1.5–1.9	1.5	1.3–1.7
No violence	1		1		1		1	
Arms/hands								
Any violence	1.4	1.3–1.6	1.4	1.3–1.6	1.7	1.5–1.9	1.5	1.3–1.8
No violence	1		1		1		1	
Neck, shoulder								
Any violence	1.4	1.3–1.6	1.5	1.3–1.6	1.6	1.4–1.8	1.4	1.2–1.6
No violence	1		1		1		1	
CV*ethnicity^d^								
Sami	–	–	1.8	1.4–2.3	2.0	1.6–2.6	1.8	1.4–2.3
Non-Sami	–	–	1.4	1.2–1.6	1.5	1.3–1.7	1.3	1.1–1.5
Back								
Any violence	1.6	1.5–1.9	1.7	1.5–1.9	1.8	1.6–2.0	1.6	1.4–1.8
No violence	1		1		1		1	
Hips, legs								
Any violence	1.5	1.3–1.7	1.5	1.3–1.7	1.7	1.5–2.0	1.5	1.3–1.8
No violence	1		1		1		1	
Head								
Any violence	1.8	1.5–2.1	1.8	1.5–2.1	1.8	1.6–2.1	1.6	1.4–1.9
No violence	1		1		1		1	
Chest								
Any violence	1.9	1.6–2.3	1.9	1.5–2.3	2.1	1.7–2.5	1.7	1.4–2.1
No violence	1		1		1		1	
Stomach/pelvic								
Any violence	1.9	1.7–2.2	1.9	1.6–2.2	2.1	1.8–2.4	1.8	1.5–2.1
No violence	1		1		1		1	
Other places								
Any violence	2.1	1.6–2.8	2.1	1.6–2.8	2.3	1.8–3.1	2.0	1.5–2.6
No violence	1		1		1		1	

Logistic regression analysis (95% CI).

a Adjusted for ethnicity,

b adjusted for ethnicity, age and education,

c adjusted for ethnicity, age, education and any other specific symptom,

d significant interaction between childhood violence (CV) and ethnicity.

**Table V:** The association between any childhood violence and chronic pain in men

			Model 1^a^		Model 2^b^		Model 3^c^	
					
Men (n=4,920)	Crude OR	CI	OR	CI	OR	CI		
Any chronic pain								
Any violence	1.5	1.3–1.7	1.5	1.3–1.7	1.6	1.5–1.9	1.5	1.3–1.7
No violence	1		1		1		1	
CV*ethnicity^a^								
Sami	–	–	1.2	0.89–1.6	1.2	0.89–1.6	1.1	0.85–1.5
Non-Sami	–	–	1.6	1.4–1.9	1.8	1.5–2.1	1.7	1.4–2.1
Arms/hands								
Any violence	1.5	1.3–1.8	–	–	1.7	1.4–2.0	1.6	1.3–1.8
No violence	1		–	–	1		1	
CV*ethnicity^d^								
Sami	–	–	1.2	0.83–1.6	1.2	0.84–1.6	1.1	0.78–1.5
Non-Sami	–	–	1.6	1.4–2.0	1.8	1.5–2.2	1.8	1.4–2.1
Neck, shoulders								
Any violence	1.5	1.3–1.7	1.5	1.3–1.7	1.6	1.3–1.9	1.5	1.3–1.8
No violence	1		1		1		1	
CV*ethnicity^d^								
Sami	–	–	1.0	0.80–1.5	1.0	0.80–1.5	1.0	0.74–1.4
Non-Sami	–	–	1.6	1.4–1.9	1.8	1.5–2.1	1.7	1.4–2.1
Back								
Any violence	1.4	1.2–1.7	1.4	1.2–1.7	1.5	1.3–1.8	1.4	1.2–1.7
No violence	1		1		1		1	
Hips, legs								
Any violence	1.6	1.3–1.8	1.6	1.3–1.8	1.7	1.4–2.0	1.6	1.3–1.9
No violence	1		1		1		1	
Head								
Any violence	2.0	1.6–2.5	2.0	1.6–2.5	2.1	1.6–2.6	1.9	1.5–2.4
No violence	1		1		1		1	
CV*ethnicity^d^								
Sami	–	–	1.3	0.76–2.1	1.3	0.72–2.1	1.1	0.67–1.9
Non-Sami	–	–	2.3	1.8–2.9	2.4	1.8–3.1	2.2	1.7–2.9
Chest								
Any violence	1.8	1.4–2.3	1.7	1.4–2.2	1.8	1.4–2.4	1.6	1.2–2.1
No violence	1		1		1		1	
Stomach/pelvic								
Any violence	1.7	1.4–2.1	1.7	1.4–2.1	1.8	1.5–2.2	1.7	1.4–2.1
No violence	1		1		1		1	
CV*ethnicity^d^								
Sami	–	–	1.1	0.73–1.7	1.1	0.73–1.7	1.0	0.67–1.6
Non-Sami	–	–	2.0	1.6–2.5				
Other places								
Any violence	2.0	1.5–2.8	2.0	1.5–2.7	2.0	1.5–2.8	1.9	1.3–2.6
No violence	1		1		1		1	
CV*ethnicity^d^								
Sami	–	–	0.92	0.47–1.8	0.92	0.46–1.8	0.79	0.39–1.6
Non-Sami	–	–	2.6	1.8–3.6	2.7	1.9–3.8	2.5	1.7–3.6

Logistic regression analysis (95% CI).

a Adjusted for ethnicity,

b adjusted for age and education,

c adjusted for age, education and any other specific symptoms,

d significant interaction between childhood violence (CV) and ethnicity.

Mean pain sites increased with any childhood violence, age, shorter education and female gender (Table VI). Poisson regression analyses revealed an increased rate ratio (RR) for pain sites of 1.4–1.5 in both Sami and non-Sami women, and non-Sami men reporting childhood violence (Table VII). In Sami men, the RR was not significantly elevated in the adjusted model (Table VII).

**Table VI:** Mean number of pain sites by gender

	All women n=6,210		All men n=4,920	
		
Chronic pain	Mean	Std. error	Mean	Std. error
Any childhood violence				
Yes	2.7	0.073	2.1	0.082
No	1.9	0.034	1.5	0.032
Ethnicity				
Sami	2.1	0.073	1.6	0.033
Non-Sami	2.1	0.035	1.7	0.075
Age				
18–34	1.3	0.057	0.89	0.061
35–49	2.0	0.054	1.4	0.053
50–69	2.6	0.048	1.9	0.043
Education				
Primary	2.7	0.099	2.1	0.079
High school	2.4	0.067	1.9	0.058
College/university	2.1	0.060	1.3	0.056
University	1.7	0.048	1.1	0.052

**Table VII:** The association between any childhood violence and number of pain sites, by gender and ethnicity

	Sami				Non-Sami			
		
Chronic pain sites	Rate ratio (RR)	CI	Adjusted RR^a^	CI	Rate ratio (RR)	CI	Adjusted RR^a^	CI
Women (n=6,210)								
Any childhood violence	1.5	1.4–1.6	1.4	1.3–1.6	1.4	1.3–1.4	1.5	1.3–1.8
Men (n=4,920)								
Any childhood violence	1.1	1.0–1.3	1.1	0.96–1.2	1.5	1.5–1.6	1.5	1.2–1.8

Poisson regression analysis.

a Adjusted for age, education and any other specific symptom.

All respondents exposed to childhood violence reported more intense pain than those not exposed to childhood violence, but the difference was not significant among Sami men. The figures among women were: Sami: 34.6% versus 20.6%, p≤0.001 and non-Sami: 31.9% versus 22.0%, p≤0.001. Among men, the figures were: Sami: 22.7% versus 18.3%, p=0.125, and non-Sami: 26.0% versus 16.6%, p≤0.001) (data not shown).

## Discussion

This study investigated the association between reported childhood violence and reported chronic pain in adulthood in a population of both Sami and non-Sami. It showed that childhood violence was associated with adult chronic pain in several pain sites of the body, as well as with an increased number of chronic pain sites and more intense pain, compared to those not exposed to childhood violence. However, among Sami men, these associations were more vague.

To our knowledge, this is the first study to investigate the association between childhood violence and adult chronic pain among an indigenous population in the Arctic, the Sami, compared to a non-Sami population. Our findings support the findings from other international studies demonstrating that chronic pain is more common in victimized persons (10,12–15). One interesting finding from our study was that for Sami men, the association between childhood violence and chronic pain in adulthood was weaker and also non-significant. One assumption is that this might be due to competing risk factors, that is, other health conditions related to chronic pain were masking the association between childhood violence and adult chronic pain among Sami men. However, the complexity of chronic pain lies in the interrelationship between physiological, psychological and sociocultural aspects (37). A more plausible explanation of the finding might be cultural differences in their interpretation of the act of violence itself, that is, the Sami men might have interpreted the violent episode(s) as less severe than non-Sami men. Such difference in cultural interpretation may be related to aspects of Sami child-rearing. An earlier study has shown a more frequent practice of physical punishment and teasing/ridiculing in Sami than in Norwegian child rearing (39). In that study, a positive correlation between physical punishment and externalizing problems emerged for the Norwegian boys, but not for the Sami boys. Teasing/ridiculing was positively correlated with internalizing problems for the Norwegian boys, but inversely correlated for the Sami boys (44). A variety of interpretations can be generated to explain this; one might be that harsh discipline has different meanings in different cultures and, hence, different outcomes. The strong impact of Sami values placed on hardiness and the endurance of hardships (41) might have heightened the threshold of tolerance for physical pain among Sami men in our study. In summary, we could argue that Sami cultural practices and values might make children less vulnerable, more resilient. Events may be recalled as violent, but be experienced as less hurting by Sami than non-Sami men.

Our finding of no ethnic differences in mean pain sites and pain located in some part of the body is in accordance with the study by Eckhoff and Kvernmo of Sami and non-Sami adolescents in Norway (31). However, their study did not measure pain located in chest, stomach and pelvic, that is, sites where our study found ethnic differences. Our finding of higher prevalence of reported stomach pain is in accordance with a study based on SAMINOR data (33). In the latter study, stomach pain was associated with milk intake. Also, our finding of higher prevalence of chest pain is in accordance with a study based on SAMINOR data, which reported a higher prevalence of angina pectoris among the Sami compared to the non-Sami (36). Furthermore, interpersonal violence has been associated with pain in the stomach and pelvic in several other studies. It might be that childhood violence may mediate some of the ethnic differences in reported chest, stomach and pelvic pain.

### Strengths of the study

The strength of this study is that our sample is from a large population-based survey, including both women and men. The large number of participants reduces the risk of a Type II error (45). The pain questions offered detailed information of pain located in various parts of the body, number of pain sites, as well as pain intensity. Hence, they provided a broad picture of chronic pain. The questions which measured interpersonal violence are a modified version of the NorVold Abuse Questionnaire (NorAQ). A validation study conducted in Sweden has shown that the abuse variables in NorAQ have showed good reliability and validity (46,47). Our modified version has previously been used in a survey on health and living conditions in Oslo in 2000–2001 (the HUBRO study) (48). Our data were collected in multiethnic municipalities, which made it possible to assess differences based on ethnicity within the same geographical areas. When classifying ethnicity, linguistic affiliation and self-identity were used as criteria. Both criteria are used by the Norwegian Sami Parliament for a register of voters. Hence, misclassification of respondents as to ethnicity may be regarded as minor. The information letter in SAMINOR 2 did not specifically address chronic pain, which makes it unlikely that participation was influenced by selection bias based on status of having chronic pain.

### Limitations of the study

Our study had a cross-sectional design which limits the potential for assessing a possible causal link between childhood violence and adult chronic pain (49). However, since this study measures experienced violence in childhood and its association with adult chronic pain, the reported exposure is likely to have taken place prior to the reported chronic pain. The overall participation rate in the SAMINOR 2 study was low, providing likely selection bias. The low participation rate indicates that the results must be interpreted with caution. We have limited information about the non-respondents. Since ethnicity is not recorded in any official register in Norway, we were not able to assess whether the proportion of the non-respondents differed in the two ethnic groups. However, a comparison between participants in the SAMINOR 2 study and those participating in the SAMINOR 1 study has been performed (43). The participation rate in SAMINOR 1 was considerably higher (60.9%) than that of this study, but the proportion of participants classified as Sami did not differ between SAMINOR 1 and SAMINOR 2. We therefore assume that the proportion of the non-respondents in SAMINOR 2 is equally distributed among the Sami and the non-Sami. Selection bias studies have shown that in assessing an association, the differences between respondents and non-respondents might be of minor importance (50). Respondents in our study differed from non-respondents as to gender, age and education. Our participants were older, were more likely to have a high education, and more women than men participated (43). Since childhood violence was associated with young age and low educational level in our study, the violence estimate might be underestimated. Hence, the strength of association would have been stronger if these groups have been included. Since our participants were older, the estimates on chronic pain might have been overestimated.

Our modified version of the NorAQ has not been validated for this particular population. Hence, differences in cultural and lingual interpretations may have influenced the observed differences in childhood violence among Sami and non-Sami respondents.

Another weakness of this study is that the respondents were asked to report experiences of childhood violence retrospectively. Recall bias is therefore possible, limiting the validity of the study. Other studies have suggested that individuals who have had painful medical conditions might have a bias in recalling earlier violence and abuse (51). This type of recall bias may overestimate the association that we found between childhood violence and adult chronic pain. On the other hand, experiences of childhood violence tend to be underreported in adulthood (5), leading to a misclassification of exposure and weakening of the association between childhood violence and adult chronic pain.

## Conclusion

This study showed that childhood violence was associated with adult chronic pain in several sites of the body regardless of ethnicity and gender. Victims of childhood violence had also an increased number of chronic pain sites, and more intense pain as adults than respondents reporting no childhood violence. However, among Sami men, this association was only significant with pain located in chest, hips/legs and back, and non-significant with increased number of chronic pain sites and higher pain intensity. Our finding may inform clinical practice. Addressing childhood violence may be included as a part of the diagnostic process for patients with unexplained chronic pain. Future studies should assess chronic pain in a longitudinal design and include information on health care utilization.
